# A case of non-HIV pneumocystis pneumonia in a patient with cirrhosis-associated immune dysfunction

**DOI:** 10.1007/s12328-025-02149-4

**Published:** 2025-06-03

**Authors:** Kai Miyamoto, Yoshihito Uchida, Shohei Tsuji, Mie Inao, Kayoko Sugawara, Masamitsu Nakao, Nobuaki Nakayama, Yukinori Imai, Suguru Mizuno, Satoshi Mochida

**Affiliations:** 1 Clinical Training Center, Saitama Medical University Hospital, Saitama, Japan; 2 Department of Gastroenterology and Hepatology, Faculty of Medicine, Saitama Medical University, Iruma-Gun, 38 Morohongo, Moroyama-cho, Saitama, 350-0495 Japan

**Keywords:** Cirrhosis-associated immune dysfunction (CAID), Pneumocystis pneumonia (PCP), Non-HIV PCP, Decompensated cirrhosis

## Abstract

A 55-year-old man with a 13-year history of alcohol-related cirrhosis was admitted for the management of refractory ascites. On day 4 of hospitalization, he developed dyspnea, and chest computed tomography (CT) revealed ground-glass opacities and reticular shadows in both lungs, predominantly in the left upper lobe. Aspiration pneumonia was initially suspected, and cefmetazole was administered. However, by day 11, his respiratory status deteriorated, with SpO₂ dropping to 90%. A follow-up chest CT showed progression of bilateral ground-glass opacities. Further testing revealed an elevated β-d-glucan level of 142.5 pg/mL and KL-6 level of 1.770 U/mL, along with a positive sputum PCR for *Pneumocystis jirovecii*, confirming a diagnosis of pneumocystis pneumonia (PCP). As human immunodeficiency virus (HIV) testing was negative, he was diagnosed with non-HIV PCP. Despite treatment with trimethoprim–sulfamethoxazole and hydrocortisone, he died on day 17. Notably, his peripheral lymphocyte count was below 500/μL before admission, suggesting that cirrhosis-associated immune dysfunction (CAID) contributed to his susceptibility to non-HIV PCP. This case highlights the importance of monitoring peripheral lymphocyte counts in patients with advanced liver cirrhosis, as CAID may increase the risk of life-threatening infections such as non-HIV PCP.

## Introduction

Patients with end-stage liver cirrhosis not only experience systemic organ dysfunction but also develop a distinct immune disorder known as cirrhosis-associated immune dysfunction (CAID) [1, 2]. CAID encompasses a broad spectrum of pathophysiological changes in cirrhosis, characterized by an imbalance between chronic systemic inflammation and immunosuppression. Chronic systemic inflammation is primarily driven by two mechanisms: (1) pathogen-associated molecular patterns (PAMPs) derived from bacterial translocation due to intestinal barrier dysfunction and (2) damage-associated molecular patterns (DAMPs) released from necrotic hepatocytes. These mechanisms contribute to persistent inflammation, which can be classified into low-grade and high-grade phases depending on severity, ultimately disrupting immune homeostasis. Conversely, cirrhosis-associated immunosuppression results from structural hepatic damage, which compromises immune surveillance and impairs immune cell function, thereby increasing susceptibility to infections [1–3]. Consequently, patients with CAID are at high risk for severe infections, including spontaneous bacterial peritonitis (SBP) and respiratory tract infections. Therefore, early diagnosis and timely treatment of infections are crucial for improving their prognosis.

*Pneumocystis pneumonia* (PCP) in the absence of human immunodeficiency virus (HIV) infection (non-HIV PCP) is an opportunistic infection caused by *Pneumocystis jirovecii*, typically affecting patients with compromised immune systems due to non-HIV-related causes. It is well documented in patients undergoing chemotherapy for malignancies, including both hematologic malignancies and solid tumors, in organ transplant recipients receiving immunosuppressive therapy, and in individuals receiving treatment for collagen vascular diseases such as granulomatosis with polyangiitis, polymyositis/dermatomyositis, or rheumatoid arthritis [4, 5]. Here, we present a case of a patient with liver cirrhosis who developed non-HIV PCP despite the absence of conventional risk factors, suggesting that CAID may have contributed to the onset of the disease.

## Case presentation

A 55-year-old man with a 13-year history of alcohol-related cirrhosis had been followed at our hospital. Despite medical advice, he continued to consume alcohol, with an estimated daily intake of approximately 60 g of pure ethanol both before and after the start of follow-up. Two years before admission, he developed refractory ascites requiring repeated paracentesis. His medications included furosemide (20 mg/day), spironolactone (25 mg/day), and tolvaptan (7.5 mg/day); however, ascites control remained inadequate. He was not receiving any immunosuppressive agents. Approximately 1 month before admission, he developed bacterial pneumonia that required antibiotic treatment. The antibiotics were administered for 5 days, after which the pneumonia was resolved.

The patient was admitted for further management of poorly controlled ascites. On admission, his SpO₂ was 100% on room air, and he had West Haven grade II hepatic encephalopathy. Physical examination revealed jaundice, a flapping tremor, a distended abdomen with a positive fluid wave, and bilateral lower-limb edema. Blood tests showed mild elevations in liver enzymes, including aspartate aminotransferase (AST) at 72 U/L, alanine aminotransferase (ALT) at 65 U/L, and γ-glutamyl transpeptidase (γ-GTP) at 56 U/L. Renal function was impaired, with a creatinine level of 2.94 mg/dL and an estimated glomerular filtration rate (eGFR) of 18.8 mL/min/1.73 m^2^, consistent with previous outpatient values. Serum albumin was 3.0 g/dL, total bilirubin was 2.9 mg/dL, and prothrombin activity was 58%, resulting in a Child–Pugh score of 11 (grade C), a Model for end-stage liver disease (MELD) score of 24, and a MELD-Na score of 30. Notably, the white blood cell (WBC) count was elevated to 14,630/μL, with 94.3% neutrophils, while the C-reactive protein (CRP) level was 0.83 mg/dL. Peripheral blood lymphocytes were markedly reduced to 263/μL. Spontaneous bacterial peritonitis was ruled out based on ascitic fluid analysis (Table 1).

**Table 1:** Blood and ascitic fluid examination at admission

Blood examination	
WBC	14,630/μL
RBC	4.74×10^6^/μL
Hemoglobin	14.9 g/dL
Hematocrit	41.1%
Platelets	107×10^4^/μL
Neutrophil	13,796 /μL
Lymphocyte	263 /μL
Total protein	5.8 g/dL
Albumin	3.0 g/dL
Creatine kinase	217 U/L
AST	72 U/L
ALT	65 U/L
LDH	486 U/L
ALP	175 U/L
γGTP	56 U/L
Total bilirubin	2.9 mg/dL
Direct bilirubin	1.3 mg/dL
Amylase	84 U/L
Creatinine	2.94 mg/dL
eGFR	18.8 mL/min/1.73 m^2^
Uric acid	9.1 mg/dL
Blood urea nitrogen	189.1 mg/dL
Ammonia	45 μg/dL
Sodium	115 mEq/L
Potassium	6.5 mEq/L
Chlorine	89 mEq/L
C-reactive protein	0.83 mg/dL
PT	58%
PT-INR	1.29
Ascitic fluid examination	
Color	Brown
Clarity	Cloudy
Specific gravity	1.011
pH	7.8
RBC	5200/μL
WBC Mononuclear cells Polymorphonuclear cells	42/μL 26/μL 16/μL
Albumin	0.3 g/dL

*ALT* alanine aminotransferase, *ALP* alkaline phosphatase, *AST* aspartate aminotransferase, *eGFR* estimated glomerular filtration rate, *γGTP* γ-glutamyl transpeptidase, *LDH* lactate dehydrogenase, *RPT* prothrombin time, *RBC* red BLOOD CELL, *WBC* white blood cell

**Fig. 1:**
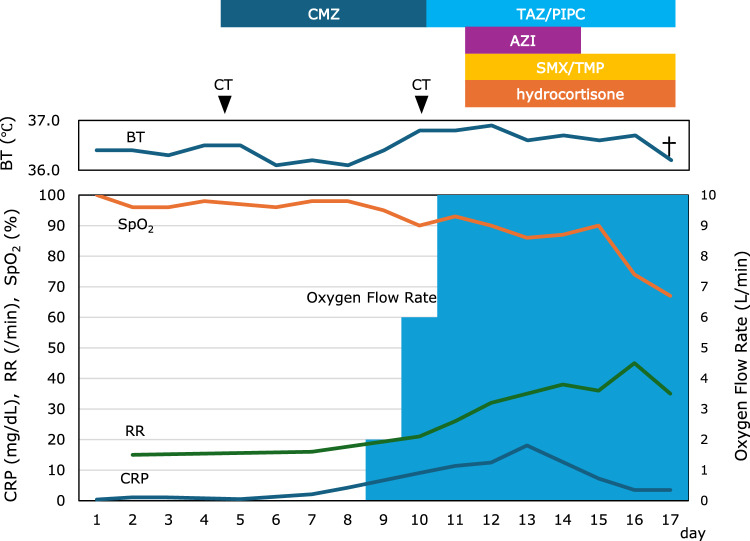
The patient’s clinical course during hospitalization. *AZI* azithromycin, *BT* body temperature, *CMZ* cefmetazole, *CRP* C-reactive protein, *RR* respiratory rate, *SMX/TMP* sulfamethoxazole/trimethoprim, *SpO*_*2*_ saturation of percutaneous Oxygen, *TAZ/PIPC* tazobactam/piperacillin

**Fig. 2:**
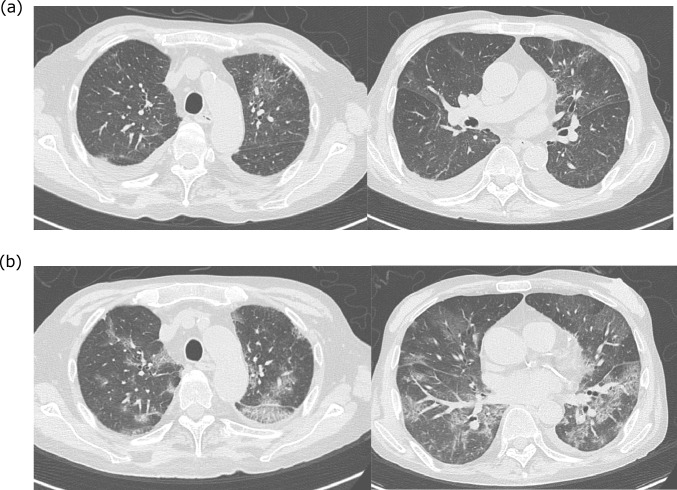
Findings from chest computed tomography (**a**) on hospital day 4 and (**b)** on hospital day 10. Bilateral ground-glass opacity and reticular opacity progressed between hospital day 4 and day 10

On day 4 of hospitalization, the patient developed new-onset dyspnea immediately after conducting upper gastrointestinal endoscopy, even though his SpO₂ remained at 97%. Throughout the clinical course, the patient did not exhibit high-grade fever or significant cough symptoms. However, blood tests showed an elevated C-reactive protein (CRP) level of 2.09 mg/dL and a white blood cell (WBC) count of 14,210/μL, with 94.6% neutrophils (Fig. 1). High-resolution computed tomography (HRCT) of the chest with a slice thickness of 1.5 mm was performed due to suspected aspiration pneumonia, which revealed ground-glass opacities and a reticular pattern in the left upper lobe (Fig. 2a). Based on these findings, bacterial pneumonia was clinically diagnosed, and cefmetazole (2.0 g/day) was initiated.

Despite antibiotic therapy, by day 10, the patient’s SpO₂ had decreased to 90%, and his CRP level had risen to 9.06 mg/dL. Supplemental oxygen therapy was initiated at 2 L/min via nasal cannula. However, his oxygen requirement rapidly increased, and by day 12, oxygen was being administered at 10 L/min via face mask (Fig. 1). A follow-up chest HRCT scan showed bilateral progression of ground-glass opacities and reticular opacities (Fig. 2b). In addition, sputum culture for acid-fast bacilli was negative, and sputum polymerase chain reaction (PCR) testing for *Mycobacterium avium*, *Mycobacterium intracellulare*, and the *Mycobacterium tuberculosis* complex was also negative. Furthermore, additional tests for infectious causes, including the FilmArray Respiratory 2.1 panel^®^ (BioMérieux, Lyon, France), were all negative (Table 2). However, despite the absence of albumin preparation administration, β-D-glucan had increased to 142.5 pg/mL, and KL-6 had risen to 1770 U/mL. Sputum PCR testing for *Pneumocystis jirovecii* was positive. Upper gastrointestinal endoscopy performed on day 4 showed no evidence of *Candida* esophagitis. Thus, the clinical diagnosis of PCP was made based on CT findings, elevated β-D-glucan, elevated KL-6, and a positive sputum PCR for *Pneumocystis jirovecii*. HIV antigen and antibody tests were both negative, confirming a clinical diagnosis of non-HIV PCP. Combination therapy with trimethoprim–sulfamethoxazole and adjunctive hydrocortisone was initiated immediately. Despite these intensive interventions, his respiratory status continued to deteriorate, and he died on day 17 of hospitalization, without the opportunity to switch from trimethoprim–sulfamethoxazole to pentamidine.

**Table 2:** Additional examinations for infectious causes

FilmArray Respiratory 2.1 panel^®^	
Adenovirus	Negative
Coronavirus 229E	Negative
Coronavirus HKU1	Negative
Coronavirus NL63	Negative
Coronavirus OC43	Negative
SARS-CoV2	Negative
Human metapneumovirus	Negative
Human rhinovirus/enterovirus	Negative
Influenza A	Negative
Influenza B	Negative
Parainfluenza virus 1	Negative
Parainfluenza virus 2	Negative
Parainfluenza virus 3	Negative
Parainfluenza virus 4	Negative
Respiratory syncytial virus	Negative
* Bordetella pertussis*	Negative
* Chlamydophila pneumoniae*	Negative
* Mycoplasma pneumoniae*	Negative
Bacterial culture	
Blood	Negative
Sputum	*Enterobacter cloacae*
Mycobacterial culture	
Sputum	Negative
Biochemical examination	
β-d-glucan	142.5 pg/mL
KL-6	1770 U/mL
HIV-1/2 antigen	Negative
HIV-1/2 antibody	Negative
PCR testing for *Pneumocystis jirovecii*	
Sputum	Positive

*HIV* human immunodeficiency virus, *PCR* polymerase chain reaction

## Discussion

The present patient developed non-HIV PCP despite the absence of conventional risk factors and experienced rapid deterioration, ultimately leading to death. Diagnosing non-HIV PCP is more challenging than diagnosing HIV-associated PCP due to the lower organism burden. While HIV-associated PCP follows a subacute to chronic course over 2 weeks–2 months, non-HIV PCP progresses rapidly, often within 1 week, leading to respiratory failure and a higher risk of severe disease. The prognosis of non-HIV PCP is also significantly worse, with a reported mortality rate of 35–50%, compared to 10–20% in HIV-associated PCP [4]. While HIV-associated PCP is strongly linked to a CD4+ T-lymphocyte count below 200/μL, non-HIV PCP can occur in patients receiving corticosteroids or other immunosuppressive therapies, even in the absence of markedly reduced lymphocyte or immunoglobulin levels [5].

Recently, Peschel.G et al. reported that 67 (44%) of 151 patients with non-HIV PCP had underlying liver cirrhosis and that liver cirrhosis itself was a poor prognostic factor for non-HIV PCP [6]. Although cases of PCP in patients with liver cirrhosis have been reported, most are associated with corticosteroid use for liver failure [7, 8]. Cases without a history of corticosteroid therapy, such as the present case, are rare. Meanwhile, Franceschini E et al. reported that among eight patients diagnosed with PCP, three had received corticosteroids, while five had no conventional risk factors [9]. These findings suggest that decompensated cirrhosis itself may lead to acquired immunodeficiency, predisposing patients to opportunistic infections (see Fig. 2).

In the present case, none of the previously identified risk factors were observed. On admission, the patient presented with marked hyponatremia (115 mEq/L), along with hepatic ascites and mild pleural effusion. Importantly, the patient’s liver cirrhosis itself was in a critically advanced stage with an extremely poor prognosis, as indicated by a MELD score of 24 and a MELD-Na score of 30. Furthermore, the patient’s peripheral blood lymphocyte count remained below 500/μL for a prolonged period during decompensated cirrhosis, indicating a state of cellular immunodeficiency similar to that seen in HIV infection.

However, due to the rapid deterioration of the patient’s clinical condition, we were unable to perform a detailed evaluation for other potential causes of immunodeficiency, such as autoimmune disease or malignant lymphoma, at the time of admission. In addition, immunological assessments including T-lymphocyte count, CD4 lymphocyte count, CD4/CD8 ratio, and gamma globulin levels were not measured. Nevertheless, given the persistent and profound lymphopenia in the context of advanced decompensated cirrhosis, without any clinical findings suggestive of alternative etiologies, we considered CAID to be the most plausible underlying cause of immunosuppression in this case.

**Fig. 3:**
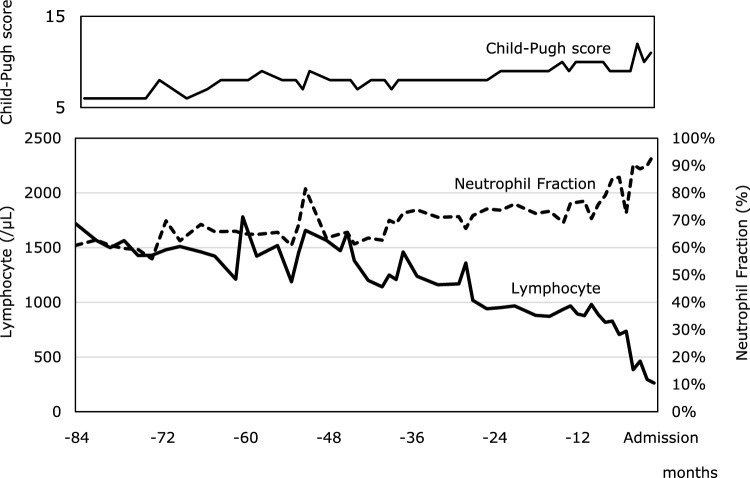
Changes in lymphocyte count and neutrophil fraction over 84 months prior to hospitalization. The thin solid line represents the Child–Pugh score, the thick solid line represents the lymphocyte count, and the dotted line represents the neutrophil fraction

In patients with liver cirrhosis, protein-energy malnutrition (PEM) and thymic atrophy lead to a decrease in peripheral lymphocyte counts, particularly T-lymphocyte counts, and further impairment of innate immunity, such as humoral immune deficiency and neutrophil dysfunction, resulting in complex immune deficiency [10–12]. Specifically, in patients with alcohol-related liver disease, persistent alcohol intake has been reported to induce profound impairment of T-cell function and dysregulated immune responses, including excessive B-cell activation, thereby contributing to immune dysfunction [13]. In addition, studies using rat models of chronic ethanol consumption have demonstrated that while the phagocytic activity of Kupffer cells is diminished, their production of cytokines and reactive oxygen species is paradoxically enhanced. These changes suggest a coexistence between impaired host defense and heightened inflammation, which may underline the increased susceptibility to infection observed in CAID [14]. In the present case, the progression of liver cirrhosis is presumed to have contributed to the reduction in lymphocyte counts and to be associated with the onset of CAID (see Fig. 3).

CAID has been recognized as a precursor to chronic decompensation and acute-on-chronic liver failure (ACLF) and is increasingly considered an important potential therapeutic target for patients with liver cirrhosis [1–3, 15]. ACLF is defined as a disease condition characterized by rapidly progressive liver failure, accompanied by functional failure of extrahepatic organs, following acute insults in patients with chronic liver disease [16]. In Japan, the primary acute insults leading to ACLF are identified as alcohol abuse and bacterial or fungal infections [17]. T-cell-mediated immunity is known to be reduced in patients with liver failure, especially in those with severe alcoholic hepatitis, who exhibit high levels of interleukin-10 and low production of interferon-γ [18, 19]. Therefore, appropriate management of CAID in patients with cirrhosis is essential to prevent infections. In particular, regular monitoring of peripheral blood lymphocyte counts is crucial for assessing the infection risk in patients with CAID. Periodic immunologic assessments can help identify high-risk individuals at an early stage, allowing for timely preventive interventions.

Furthermore, maintaining the balance of the gut microbiota and implementing nutritional management strategies may help suppress the progression of CAID. Recent studies have shown that gut dysbiosis promotes chronic inflammation and immune dysregulation, thereby increasing the risk of infection in patients with cirrhosis [20, 21]. Therefore, therapeutic strategies such as modulation of the gut microbiota with probiotics or prebiotics, correction of hypoalbuminemia with nutritional support—including the use of branched-chain amino acids and dietary sodium restriction—may be useful in preventing infections.

Despite its clinical importance, CAID lacks a universally accepted definition and established diagnostic criteria. Therefore, its pathophysiology must be inferred from its clinical course. A comprehensive analysis of serological markers, including various cytokines and chemokines, is essential to clarify the underlying mechanisms [1, 2]. Within this complex immunological background, Nakamura et al. proposed that a decrease in peripheral blood lymphocyte count is an independent prognostic factor for patients with liver cirrhosis, along with the presence of hepatocellular carcinoma (HCC) and a high MELD score. They introduced a staging system for CAID based on two parameters: a peripheral blood lymphocyte count below 1500/μL and a neutrophil fraction of 70% or higher. Specifically, they classified patients as follows: Stage 0 for those with a lymphocyte count of 1500/μL or more, Stage 1 for those with a lymphocyte count below 1500/μL and a neutrophil fraction below 70%, and Stage 2 for those with a lymphocyte count below 1500/μL and a neutrophil fraction of 70% or higher. This classification demonstrated significant prognostic stratification among patients with liver cirrhosis [22].

In the present case, until 44 months prior to admission, the patient maintained a white blood cell count of approximately 5000–7000/µL, with a neutrophil fraction below 70% and a lymphocyte count above 1500/µL. However, the lymphocyte count gradually declined, and by 24 months prior to admission, the neutrophil fraction exceeded 70% while the lymphocyte count fell below 1000/µL, suggesting both the development of systemic chronic inflammation and an immunosuppressive state. Subsequently, the lymphocyte count continued to decrease, dropping below 500/µL 2 months before admission. This persistent lymphopenia indicates the progression of immunosuppression, ultimately leading to the development of PCP. Moreover, the occurrence of bacterial pneumonia prior to admission further underscores the possibility that the patient’s immune function had already been significantly compromised by the progression of CAID.

This case highlights the critical role of CAID in the development of serious opportunistic infections, including non-HIV PCP. As CAID represents a continuum between chronic systemic inflammation and immunosuppression, understanding its impact is crucial for early detection and risk stratification in patients with cirrhosis. While current management focuses primarily on infection prevention and supportive care, future research should explore targeted immunomodulatory therapies to restore immune balance without exacerbating systemic inflammation. In addition, prospective studies evaluating CAID as a prognostic tool could help refine screening strategies and guide personalized interventions for patients at high risk of life-threatening infections and ACLF. In conclusion, in patients with advanced liver cirrhosis, a decline in peripheral blood lymphocyte count serves as an important indicator of infection risk, underscoring the value of prevention, early diagnosis, and prompt treatment.

## Abbreviations

ACLF: Acute-on-chronic liver failure
ALT: Alanine aminotransferase
AST: Aspartate aminotransferase
BCAA: Branched-chain amino acids
CAID: Cirrhosis-associated immune dysfunction
CRP: C-reactive protein
CT: Computed tomography
DAMPs: Damage-associated molecular patterns
γ-GTP: Gamma-glutamyl transferase
HCC: Hepatocellular carcinoma
HIV: Human immunodeficiency virus
KL-6: Krebs von den lungen-6
MELD: Model for end-stage liver disease
PAMPs: Pathogen-associated molecular patterns
PCP: Pneumocystis pneumonia
PCR: Polymerase chain reaction
SBP: Spontaneous bacterial peritonitis
SpO₂: Peripheral capillary oxygen saturation
WBC: White blood cell
